# Antifungal immunity mediated by C-type lectin receptors may be a novel target in immunotherapy for urothelial bladder cancer

**DOI:** 10.3389/fimmu.2022.911325

**Published:** 2022-09-05

**Authors:** Tianhang Li, Tianyao Liu, Zihan Zhao, Yuchen Pan, Xinyan Xu, Yulin Zhang, Shoubin Zhan, Shengkai Zhou, Wenjie Zhu, Hongqian Guo, Rong Yang

**Affiliations:** ^1^ Department of Urology, Affiliated Nanjing Drum Tower Hospital, Medical School, Nanjing University, Nanjing, China; ^2^ State Key Laboratory of Pharmaceutical Biotechnology, Division of Immunology, Medical School, Nanjing University, Nanjing, China; ^3^ Jiangsu Engineering Research Center for microRNA Biology and Biotechnology, State Key Laboratory of Pharmaceutical Biotechnology, School of Life Sciences, Nanjing University, Nanjing, China

**Keywords:** mycobiome, bladder cancer, immunity, C-type lectin receptor, pattern recognition receptor

## Abstract

Immunotherapies, such as immune-checkpoint blockade and adoptive T-cell therapy, offer novel treatment options with good efficacy for patients with urothelial bladder cancer. However, heterogeneity and therapeutic resistance have limited the use of immunotherapy. Further research into immune-regulatory mechanisms in bladder cancer is urgently required. Emerging evidence demonstrates that the commensal microbiota and its interactions with host immunity play pivotal roles in a variety of physiological and pathological processes, including in cancer. The gut microbiota has been identified as a potentially effective target of treatment that can be synergized with immunotherapy. The urothelial tract is also a key site for multiple microbes, although the immune-regulatory role of the urinary microbiome in the process of carcinogenesis of bladder cancer remains to be elucidated. We performed a comprehensive analysis of the expression and biological functions of C-type lectin receptors (CLRs), which have been recognized as innate pathogen-associated receptors for fungal microbiota, in bladder cancer. In line with previous research on fungal colonization of the urothelial tract, we found that CLRs, including Dectin-1, Dectin-2, Dectin-3, and macrophage-inducible Ca^2+^-dependent lectin receptor (Mincle), had a significant association with immune infiltration in bladder cancer. Multiple innate and adaptive pathways are positively correlated with the upregulation of CLRs. In addition, we found a significant correlation between the expression of CLRs and a range of immune-checkpoint proteins in bladder cancer. Based on previous studies and our findings, we hypothesize that the urinary mycobiome plays a key role in the pathogenesis of bladder cancer and call for more research on CLR-mediated anti-fungal immunity against bladder cancer as a novel target for immunotherapy in urothelial bladder cancer.

## Introduction

Bladder cancer continues to be one of the most common urological malignancies worldwide, with the highest incidence and mortality rates (1–3). Several therapeutic approaches, such as transurethral resection of bladder tumor (TUR-BT) and intravesical infusion chemotherapy (4, 5), are available in early-stage bladder cancer. Where bladder cancer progresses to the muscle-invasive stage, the incidence rate of lymph node metastasis increases significantly and the survival rate decreases significantly (6, 7). Radical cystectomy is an effective treatment option for patients with advanced bladder cancer. However, excision of the bladder can lead to poor quality of life and the possibility of recurrence remains, even under the maintenance of adjuvant chemotherapy (8). Recently, adjuvant immunotherapy with immune-checkpoint blockade agents has been reported to have great efficacy in the treatment of bladder cancer and bladder retention in a range of clinical trials (9–11). Nevertheless, high levels of efficacy and heterogeneity and the frequent occurrence of immune resistance limit the benefits of immunotherapy. Even though patients can respond well to immune checkpoint blockade (ICB) therapy, with prolonged survival, most patients do not benefit from ICB therapy. In addition, even those who initially respond well to ICB therapy can lose their primary sensitivity to the therapy and bladder cancer can develop adaptive immune resistance (12, 13). Therefore, clinicians and researchers are trying to understand the immune-regulatory mechanisms in the tumor microenvironment and develop therapies with novel targets that can be synergizing with immunotherapy.

The commensal microbiota, which refers to the colonizing microbes, such as bacteria, fungi, archaea, and their metabolites, in different organs of our body, has been revealed recently as a pivotal player in our immune system (14, 15). The best-studied microbiome is the gut microbiota, which has been found to exert powerful immune functions by interacting with various receptors expressed on immune cells (16–18). Multiple gastrointestinal pathological processes, including inflammatory bowel disease, gastric carcinoma, and colon cancer, have been found to be associated with specific alterations of the gut microbiota and abnormal activation of immune responses (16, 19–21). Notably, the regulatory networks mediated by the gut microbiota have even been extended to further organs, referred to as the gut–brain axis (22) and the gut–lung axis (23). The remote regulations performed by the gut microbiota could participate in the disease progression of depression and lung cancer (24–26). Overall, the commensal microbiome, especially the gut microbiome, plays powerful and complex roles in our physiological system. The commensal fungi, also known as the mycobiome, has long been ignored or mistaken to be an unimportant bystander in the gut microbiota. However, in recent studies, researchers have identified that the fungal microbiota plays crucial roles in a range of cancers (27–29). Anti-fungal immunity in our mucosa is mediated mainly by C-type lectin receptors (CLRs), which represent a family of transmembrane receptors recognizing endogenous or exogenous ligands mainly derived from fungi. Immune responses can be activated following the recognition of fungi. The four main members of the CLR family include Dectin-1, Dectin-2, Dectin-3, and macrophage-inducible Ca^2+^-dependent lectin receptor (Mincle). These differ in the extracellular domain, recognized ligands, and downstream activation pathways (30–32). Dectin-3 has been found to serve as an immune barrier against the potential invasion of *Candida tropicalis*, and its deficiency can lead to impaired anti-fungal immunity and subsequent colitis (33). Furthermore, Dectin-3 has also been found to participate in the tumorigenesis of colon cancer through interaction with *Candida albicans*, thus forming a crosstalk network between innate immune cells and tumor cells (34). By contrast, the activation of Dectin-1 by galectin-9 on tumor-infiltrating macrophages has been found to induce immune escape and accelerate the progression of pancreatic cancer, which suggests a dual function of CLRs in cancer (35).

The idea that the urinary tract, including the bladder, is sterile has dominated our thinking for a long time. However, with the development of advanced detection technology, multiple studies have shown the importance of the urinary microbiome, which may provide novel targets and strategies in the treatment of various urological diseases, including bladder cancer (36, 37). The mycobiome has also been identified as a widespread microorganism with high levels of diversity and heterogeneity among urinary systems, and it may assume key functions during the process of bladder cancer (38). Nevertheless, related studies are lacking. In this article, we report our comprehensive analysis of the correlations between the expression, function, and immune infiltration of CLRs in bladder cancer to explore the possible mechanisms of immune-regulatory functions exerted by the urinary mycobiome and CLR-expressing immune cells. Furthermore, we discuss the possibility and feasibility of utilizing CLR-mediated anti-fungal immunity as a novel effective target for synergizing and optimizing the efficacy of immune-checkpoint blockade therapy in bladder cancer.

## Justification of the hypothesis

### The existence of and alterations in the urinary microbiome in pathological conditions

An important premise for our hypothesis is the widespread presence of the mycobiome in the urinary system, especially in the bladder. For a long time, the urinary tract has been considered to be a sterile organ unless it is in an infected state. However, with the rapid development of culture technology and molecular sequencing methods, several studies have been carried out to elucidate the potential functional microbes in the tumorigenesis of bladder cancer. Significant overexpression of *Streptococcus* has been found in urothelial cancer patients, suggesting an association between a specific type of bacteria and bladder cancer (39). Moreover, the utilization of the bacillus Calmette–Guérin (BCG) vaccine, which involves the administration of *Mycobacterium tuberculosis* with attenuated toxicity, has become the standard therapeutic approach for non-muscle-invasive or intermediate-risk bladder cancer (4). Although the exact mechanisms are still unclear, the current consensus is that the effectiveness of the vaccine is dependent on the inflammatory immune responses provoked by the induction of this specific bacteria, which results in the anti-tumor effect. Two questions emerge from this long-standing therapeutic application: (1) Would the input of new bacteria alter the microbiome in the bladder and influence immunity? Previous studies have transplanted microbiota and found that the transfer of intestinal microbiota or the mono-colonization of the mycobiome can reshape the composition of the gut microbiota and regulate anti-cancer immunity; similar effects may be found in the bladder microbiota (40, 41). (2) Do any other exogenous or endogenous microbes possess similar immune-activating characteristics? To address these questions, we may need to first identify the specific microbiota in the bladder under different conditions.

A series of studies have been carried out to explore the commensal microbiota in the bladder of healthy hosts. The most frequently detected bacteria genera were *Streptococcus* and *Lactobacillus* (42). Other bacteria have been found less frequently in healthy bladders, such as *Veillonella*, *Burkholderia*, *Alloscardovia*, and *Saccharofermentans* (36, 37). Nevertheless, observation of the commensal urinary microbiome has lacked sufficient accuracy owing to the technical limitations of 16S ribosomal RNA (rRNA) sequencing, which has not been able to differentiate between dead, living and ruptured bacteria. In addition, vulvovaginal contamination has affected detection accuracy in women. Interestingly, the urinary microbiota is not a static symbiont and has shown great diversity according to age and sex (36, 43), which indicates that in one individual the urinary microbiome can experience active reprogramming in the regulation of various factors, suggesting possible regulatory functions of the urinary microbiota under pathological conditions.

**Figure 1:**
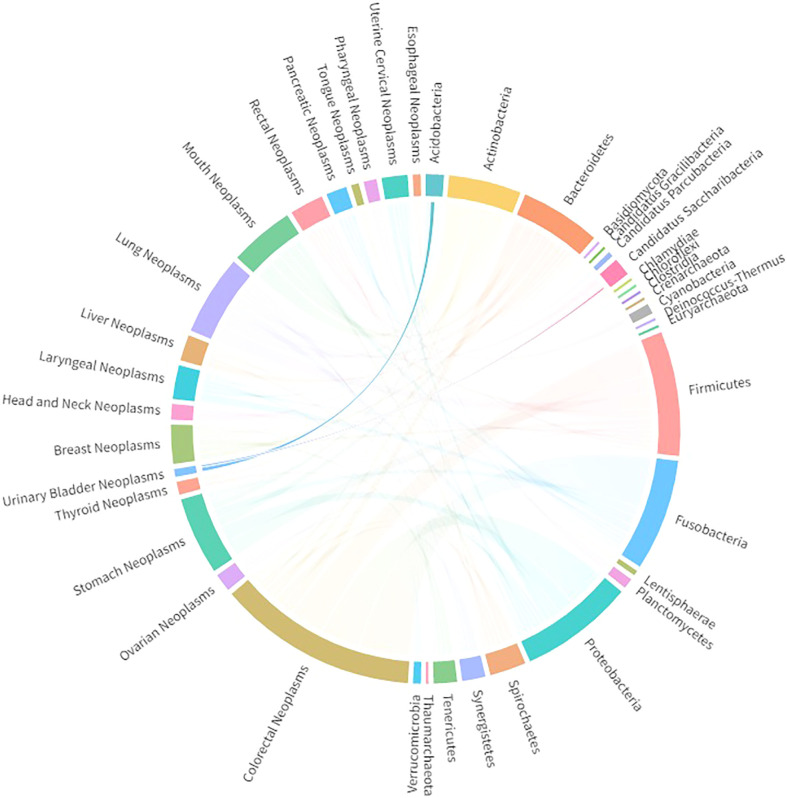
Significantly associated bacteria phyla detected in bladder cancer patients, extracted from DIANA Lab databases.

As alluded to above, different anatomical structures appear to result in differences in the urinary microbiota of men and women. Taking into consideration the significant difference in the incidence rate of bladder cancer among men and women, the microbiota has emerged as another possible factor, alongside hormonal factors, in bladder cancer tumorigenesis. Multiple studies have been conducted to identify abundant or dominant microbiota in the bladder tissue of bladder cancer patients, and results vary. In some studies, members of the phylum Firmicutes have been found to be the most abundant bacteria in the urinary and bladder tissue of bladder cancer patients (44, 45), whereas in other studies the dominant phylum has been reported to be Proteobacteria (46, 47). Cyanobacteria have been detected in bladder cancer patients’ urine and tissues, constituting up to 8% of the urinary microbiome (45, 48). It is worth noting that this phylum of bacteria can produce toxic microcystins, which have been found to promote the progression of colorectal and liver cancer (49, 50), but it is still unclear whether Cyanobacteria can have a similar function in the development of bladder cancer. Public databases from DIANA Lab (University of Thessaly, Thessaly, Greece; www.dianalab.gr) were utilized to explore the bacteria phyla significantly associated with bladder cancer. As shown in **Figure 1**, Acidobacteria and Candidatus Saccharibacteria are the two bacteria phyla that are most significantly altered in urothelial bladder cancer patients compared with healthy subjects. However, the data lack sufficient stability; for example, one study found that Acidobacteria levels were higher in the urine of bladder cancer patients than in the urine of control participants, whereas another study found the opposite (see DIANA Lab data). Therefore, taking the presently available studies into account, we could come to several conclusions. First, the commensal microbiota is a widespread, symbiotic organism residing in our urinary system, particularly in the bladder. However, microbiota characteristics are highly heterogeneous among individuals, at least at the taxonomic level of the kingdom. Although several studies have been conducted to identify the dominant or functional bacteria in the tumorigenesis of bladder cancer, we are still far from a definitive answer. Therefore, alongside more research on bacteria, it may also be necessary to do more research on other microorganisms.

### Mycobiome and C-type lectin receptor-mediated anti-fungal immunity in the context of cancer

CLRs, defined as a family of receptors characterized by the C-type lectin domain, are the major pattern recognition receptors for detecting fungi. By recognizing β-glucans, α-mannan, etc., which are the major components expressed on the cell walls of fungi, CLRs can initiate innate as well as adaptive immune responses through activation of a range of downstream signaling pathways (32). Major CLRs include Dectin-1, Dectin-2, Dectin-3, and Mincle, which are expressed mainly on myeloid immune cells (51, 52). By regulating anti-fungal immunity, various CLRs have been found to participate in multiple pathological immune processes, including colitis (33, 53), Crohn’s disease (54, 55), and systemic lupus erythematosus (56). More importantly, CLR–fungi crosstalk has been identified as a major carcinogenic factor. Zhu et al. (34) found that Dectin-3 deficiency can increase the fungal burden of *C. albicans*, which in turn can reprogram innate immune cells metabolically and enhance the tumorigenesis of colon cancer. Furthermore, another study found that macrophage-expressed Dectin-1 can accelerate the progression of pancreatic cancer through ligation with galectin-9 in the tumor microenvironment, which depends on adaptive immunosuppression (35). This study reminds us that CLRs can also exert immune functions by interacting with non-pathogen ligands in the tumor microenvironment. Nevertheless, the roles played by CLRs in the context of bladder cancer and whether CLR–fungi crosstalk can have crucial functions in the tumorigenesis of bladder cancer are still unclear to us. To clarify this, we conducted a comprehensive analysis of CLRs and their related biological processes in bladder cancer. Specifically, when it comes to the signaling mechanism and immune functions, CLRs can be divided into two subgroups according to different intracellular signaling motifs: those associated with the immunoreceptor tyrosine-based activation motif (ITAM) domain and those associated with non-ITAM domains (57). The activation of Dectin-1 triggers the intracellular signaling pathways through the direct transduction of the ITAM-like motif(s) within the cytoplasmic tails; Dectin-2, Dectin-3, and Mincle transduce the signaling pathways indirectly by ITAM-containing Fc receptor γ (FcR-γ) chains (58, 59). On recognition of the ligand, the receptors are activated and Src kinases are recruited to induce tyrosine phosphorylation of the ITAM motif, thus further activating SYK kinase and thereby activating the downstream signaling pathways (58, 60). Following its activation, SYK kinase induces the phosphorylation of protein kinase C-δ (PKC-δ), which then mediates the phosphorylation of caspase recruitment domain-containing protein 9 (CARD9). This process leads to the subsequent formation of the complex of CARD9 along with B-cell lymphoma/leukemia 10 (Bcl-10) and mucosa-associated lymphoid tissue lymphoma translocation protein 1 (MALT1) (61), which, finally, triggers the activation of nuclear factor kappa-light-chain-enhancer of activated B cells (NF-κB) (62) or extracellular signal-regulated kinases (ERKs) (63). In addition to the SYK-dominated signaling pathway, Dectin-1 can also activate another signaling pathway independent of SYK kinase, which is mainly mediated by Raf-1 (61). Following the activation of these signaling pathways, multiple signaling events are induced, including respiratory burst, phagocytosis, and the production of a range of inflammatory mediators (51, 64, 65). Through secretion of interleukin 1 beta (IL-1β), interleukin 6 (IL-6), and interleukin 23 (IL-23), the cluster of differentiation 4+ (CD4+) T cells would be polarized into T helper type 1 (Th1)/T helper type 17 (Th17) cells, forming the essential anti-fungal immunity (61, 66).

In addition to the four major CLRs we have introduced here, there are many other CLRs, such as MR (CD206), DC-SIGN (CD209a), CD23, and CR3. Therefore, the reader may wonder why only these four CLRs are discussed here. The reasons can be divided into two categories. First, the four CLRs discussed here (i.e. Dectin-1, Dectin-2, Dectin-3, and Mincle) are the best-studied CLRs that recognize fungal ligands and exert crucial anti-fungal immune functions (52). Importantly, owing to the anatomical and biological features of the bladder and bladder cancer, we need to focus on the CLRs that play well-studied regulatory roles on mucosal immunity. These CLRs are the most commonly recognized receptors exerting such functions (33, 67–69). Therefore, we selected these four CLRs as crucial mediators in anti-fungal immunity in the bladder and potential regulators in the tumorigenesis of bladder cancer. Second, there is insufficient evidence of the capability of other CLRs to modulate immune-regulatory functions in the bladder. Although one study found that MR may transduce downstream signaling pathways upon *M. tuberculosis* infection (70), further investigation concluded that MR has no significant impact on the host’s anti-fungal immunity (71, 72), further indicating that MR may not be a major receptor in anti-fungal immunity. Compared with MR, DC-SIGN has been found to perform a more important role in anti-fungal immunity, with its pivotal fungi recognition ability (73–75). Nevertheless, to our knowledge no gene-targeting experiment has been carried out to evaluate the importance of DC-SIGN in anti-fungal immunity. Similarly, the exact functional mechanisms of other CLRs are still far from clear. Therefore, limited by the relatively small number of studies of other CLRs, we focused on the four CLRs that are well studied and recognized as the major CLRs in the homeostasis and pathology of the bladder. Nevertheless, other CLRs may play undiscovered roles in the pathogenesis of bladder diseases, especially bladder cancer, and future investigation of the underlying molecular mechanisms of crosstalk among other fungal pattern recognition receptors (PRRs) will be of great significance. Taken together, the four CLRs we discuss here (i.e. Dectin-1, Dectin-2, Dectin-3, and Mincle) are major regulators in anti-fungal immunity in bladder cancer and deserve further investigation and discussion.

Notably, although CLRs are commonly recognized as major sensors in fungi recognition, CLRs may also recognize other pathogens, such as bacteria, viruses, and fungi, leading to the induction of immune responses. As is shown in **Figure 1**, two bacterial phyla have been found to be significantly altered in urothelial bladder cancer patients. In addition, as discussed above, Firmicutes, Proteobacteria, and Cyanobacteria have been found to be abundant in the urinary samples of bladder cancer patients (45–48). Therefore, there could also be associations between CLRs and bladder cancer-related bacteria. Nagata et al. (76) found that the metabolites derived from *Helicobacter pylori* can be recognized by Mincle, inducing T-cell inflammatory responses and leading to gastritis. More recently, an obligately intracellular bacteria, *Orientia tsutsugamushi*, was found to be able to upregulate the expression of Mincle on macrophages, which subsequently triggers sustained inflammatory responses (77), indicating that specific bacteria may also modulate CLRs expression and thereby induce downstream immune functions. Therefore, an abundance of certain bacteria may be another contributing factor in CLR expression and function in bladder cancer.

The mycobiome, which refers to the fungal microbiota, is another major component in our microbial ecosystem. However, the importance of the mycobiome for our systemic homeostasis and various pathological processes has long been underestimated. Recently, the mycobiome and mucosal anti-fungal immunity have been found to be influential in a range of diseases (78). As the largest shelter for microorganisms, the gastrointestinal tract was the first to be studied. *C. albicans* was found to activate macrophage inflammatory responses through β-glucan exposure, which enhances the pathological process of inflammatory bowel disease (79). Similarly, *C. tropicalis* was found to participate in the formation of colonic colitis through interaction with Dectin-3-expressing macrophages (33). These studies suggest that the role played by the commensal fungi in immune homeostasis is primarily in the induction of innate immunity. Moreover, fungi have been identified as influential in carcinogenesis. Commensal *C. tropicalis* was found to increase myeloid-derived suppressor cell (MDSC) infiltration in CARD9 deficiency, contributing to the development of colon cancer (40, 80). Furthermore, *C. albicans* was found to promote the progression of colon cancer through metabolic regulation of innate immune cells (34, 81). These studies demonstrate the significant contribution of commensal fungi in colon carcinogenesis, which has so far been neglected.

When it comes to the bladder, the role of the commensal mycobiome is still unclear, limited as we are by the lack of comprehensive studies identifying commensal fungi. However, there exist a few previous studies of the urinary mycobiome, which may provide further clues. *Candida* spp. have been detected in urinary samples from healthy control participants as well as patients with urological disorders (82–84). More recently, through the application of next-generation sequencing (NGS) with internal transcribed spacer 1 (ITS1)-region amplification, researchers found significant diversity in the fungal population among individuals (38). Previous studies have identified Saccharomycetes fungi, including *Saccharomyces* and *Candida* spp., in the urine of patients with urological symptoms (85, 86), which suggests that these fungi may play a role in our urinary system. In the context of bladder cancer, we have identified the potential regulatory roles of different bacteria taxa in tumorigenesis (87). Therefore, we may wonder whether there exist specific pathogenic fungi in bladder cancer. Aykut et al. (88) utilized the 18S internal transcribed spacer (ITS) sequencing approach and found an abundance of *Malassezia* in the pancreas and pancreatic cancer tissue, further elucidating its oncogenic function through mannose-binding lectin **(**MBL) activation. Moreover, another study involving 18S rRNA sequencing found a predominant abundance of *Malassezia* in the fungal mycobiome in pancreatic cancer tumor tissues. Notably, it was found that the intratumoral mycobiome can promote the production of IL-33 from cancer cells and subsequently enhance the recruitment and activation of Th2 cells and innate lymphoid cells in the microenvironment of pancreatic cancer, thus inhibiting the progression of the tumor (89). In addition, the role of the fungal mycobiome in tumorigenesis in colon cancer has been demonstrated (40, 80). These studies indicate that the tumor-resident mycobiome can be detected precisely using NGS technology. Back in the 1990s, Skoutelis et al. (90) performed an analysis comparing the adherence of urine-derived *C. albicans* to bladder epithelial cells between bladder cancer patients and control participants and found a significant increase in *C. albicans* adherence among bladder cancer patients, which suggests that the bladder urothelium in such patients may exhibit specific alterations and thus be more susceptible to fungal colonization. Furthermore, NGS with ITS1 amplification has been utilized to identify the rich and heterogeneous fungal mycobiome in multiple urinary samples (91). In addition, several case reports have reported the colonization of the bladder by certain fungal species in the context of cancer (92) or diabetic neurogenic bladder (93). Although the utilization of 16S rRNA sequencing technology has helped us to identify specifically bladder cancer-related bacteria microbiota, the bladder cancer-related mycobiome is still not understood. Related studies of the participation of the commensal fungi in bladder cancer carcinogenesis are still lacking. Therefore, deeper insights into the mycobiome in the bladder and its interactions with the host are warranted in the future.

### Comprehensive analysis of CLRs and related immunity in bladder cancer

#### Expression of C-type lectin receptors in bladder cancer tissue and normal tissue

**Figure 2:**
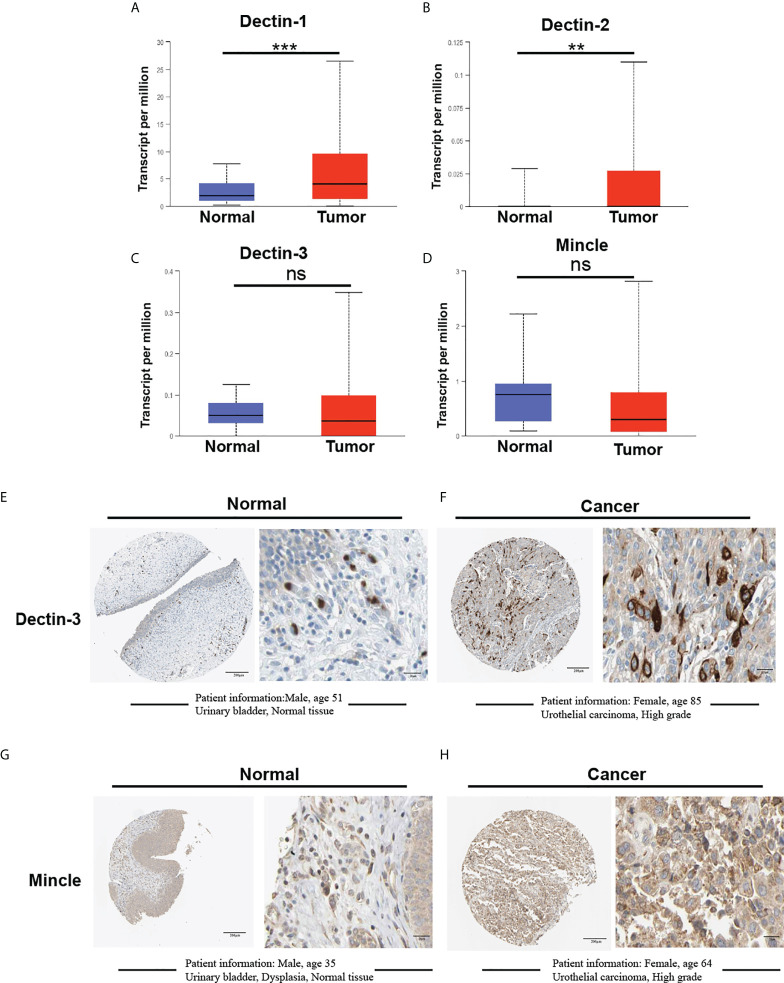
Comparative analysis of mRNA expression of CLRs between bladder cancer tumor tissue and normal bladder tissues using the Cancer Genome Atlas. (TCGA; https://portal.gdc.cancer.gov) database **(A–D)**. The TCGA database is part of a landmark cancer genomics program that has characterized over 20,000 primary cancers of 33 types and corresponding normal tissue samples. Our analysis enrolled 19 normal tissues and 408 tumor tissues from the bladder cancer database. IHC results show the protein expression of Dectin-3 and Mincle in normal bladder tissues **(E, G)** and bladder cancer tumor tissues **(F, H)**. The basic clinical information of the enrolled patients is presented. ns means no significant difference was found between the two groups.**p < 0.01,***p < 0.001.

The messenger RNA (mRNA) expression of the four CLRs in bladder cancer primary tumor tissues and normal tissues was first evaluated using a sample comprising 408 primary tumor tissues and 19 normal bladder tissues. Dectin-1 was significantly upregulated in tumor tissue compared with normal tissues (**Figure 2A**). Similar results were observed for Dectin-2 (*p* < 0.01), but Dectin-2 transcriptional levels were low in both normal and tumor tissues (**Figure 2B**). As with Dectin-3 and Mincle, no significant differences in expression were found between normal tissues and tumor tissues (**Figures 2C, D**). To further detect the expression location and status at the protein level, we retrieved immunohistochemical (IHC) data (only Dectin-3 and Mincle were available) from the Human Protein Atlas (HPA) database (Kungliga Tekniska högskolan Royal Institute of Technology, Stockholm, Sweden; www.proteinatlas.org). As shown in **Figures 2E, G**, there was a relatively low but extensive expression of Dectin-3 and Mincle in urothelial cells in the urothelial mucosal layer of the bladder. In addition, both Dectin-3 and Mincle were observed to be expressed in the infiltrated immune cells in the submucosa. In bladder cancer tumor tissue, in both the tumor cells and the tumor-infiltrated immune cells, both high and low expressive levels of Dectin-3 and Mincle were observed (**Figures 2F, H**).

#### Gene enrichment and potential pathways analysis

**Figure 3:**
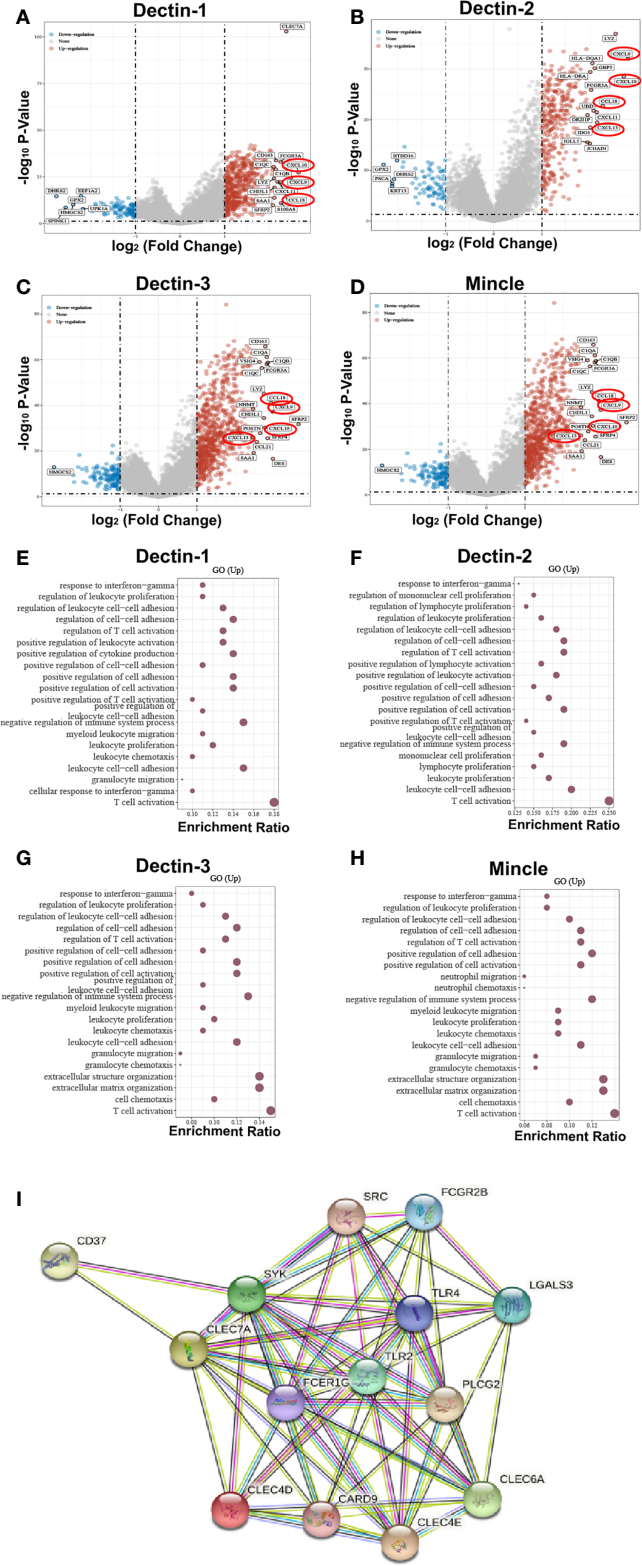
DEGs were found in bladder cancer patients in the CLR-high and CLR-low groups **(A–D)**. The CXC subfamily chemokines of CXCL9, CXCL10, and CXCL13, and the CC subfamily chemokine of CCL18 were found to be consistently expressed in bladder cancer patients in the CLR-high group, and levels of expression were significantly higher among bladder cancer patients in the CLR-high group than among the those in the CLR-low group. CLR gene enrichment pathway prediction based on the DEGs in bladder cancer patients **(E–H)**. Protein crosstalk networks centered on CLRs using STRING **(I)**. Toll-like receptor 2 (TLR2), toll-like receptor 4 (TLR4), and Fc epsilon receptor Ig (FCER1G) are the predicted functioning partners with CLRs.

**Figure 4:**
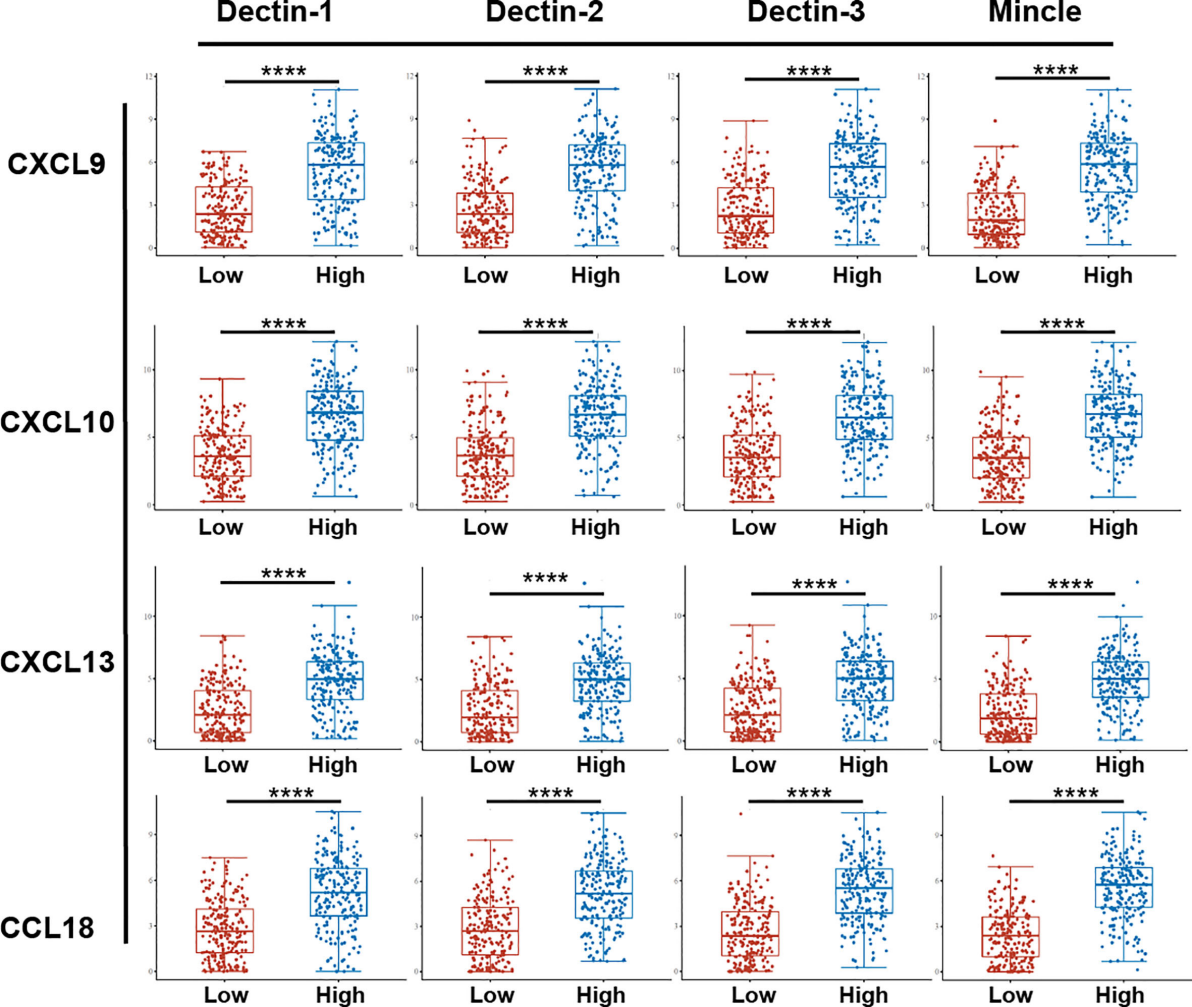
Comparison of specific chemokines expression between bladder cancer patients in the CLR-low and CLR-high groups. The 406 bladder cancer patients from the TCGA database were divided into CLR-low (*n* = 203) and CLR-high (*n* = 203) groups according to whether the expression of CLR mRNA was above or below the median. The abscissa represents different sample groups and the ordinate represents the distribution of expression of CLRs; different colors represent different groups. *p < 0.05, **p < 0.01,***p < 0.001, ****p < 0.0001. The statistical differences between the two groups were compared using a Wilcox test.

To further explore the biological functions and regulatory roles performed by CLRs in the tumor microenvironment, we performed a differential gene analysis based on TCGA data from bladder cancer tissue samples, which can be divided into two subgroups, CLR-high and CLR-low, to determine the differentially expressed genes (DEGs) potentially under the regulation of CLRs in bladder cancer (**Figures 3A–D**). The results show that the most common DEGs include those in the C-X-C motif chemokine ligand (CXCL) family and the chemokine ligand (CCL) family, which largely function as immune-regulatory chemokines involved in the induction and chemotaxis of immune cells. To further compare the expression of the genes of interest in the CLR-low and CLR-high groups, we also performed specific group comparisons for CXCL9, CXCL10, CXCL13, and CCL18 (**Figure 4**). Other DEGs may also indicate specific downstream biological functions of different CLRs in bladder cancer. To further explore the potential cellular pathways mediated by CLRs in bladder cancer, we performed a gene ontology (GO) enrichment pathway prediction analysis based on the previously obtained differentiated genes (**Figures 3E–H**). Interestingly, we found that these four CLRs shared similar pathways, which focused on the positive regulation of the activation and migration of innate and adaptive immunity, which suggests that CLRs potentially provide immunity against bladder cancer. In addition, we evaluated the interaction network among CLRs and their work partner proteins using STRING (Search Tool for the Retrieval of Interacting Genes/Proteins; ELIXER, Hinxton, UK; https://string-db.org) (**Figure 3I**).

#### Associations between C-type lectin receptors with immune cells and immune-checkpoint proteins

**Figure 5:**
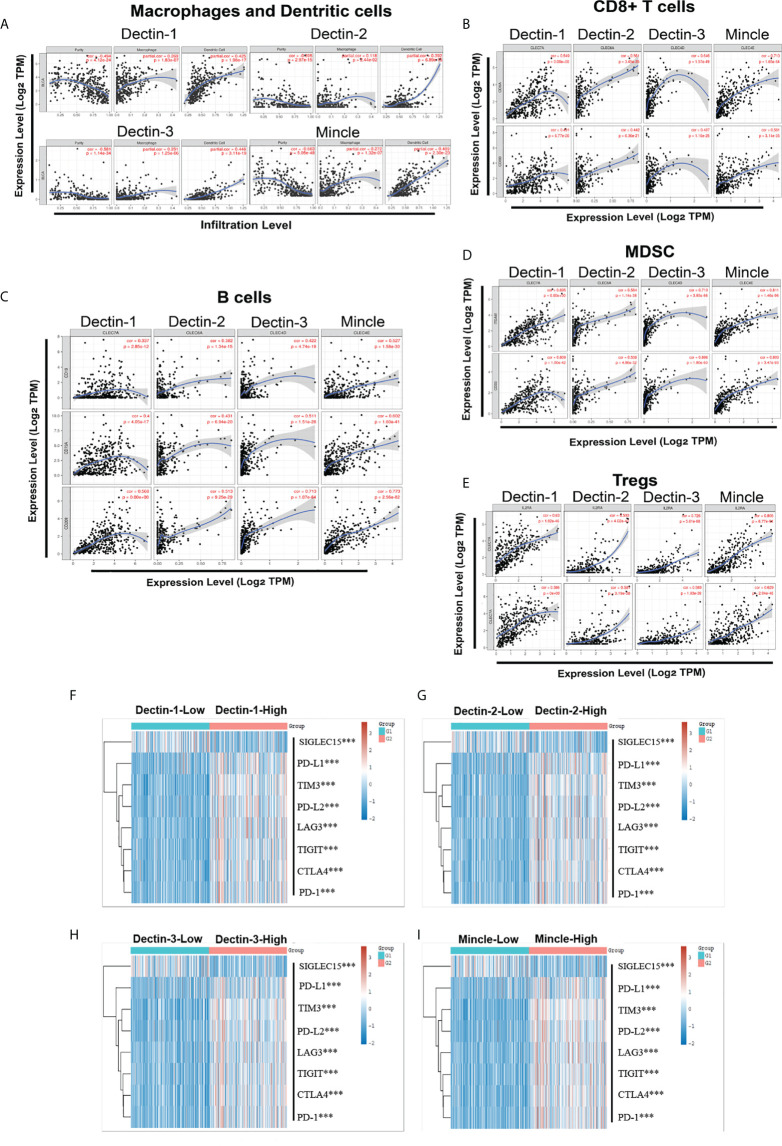
Association between CLR expression and multiple crucial immune cell infiltration in bladder cancer, including macrophages, neutrophils, and dendritic cells **(A)**, CD8+ T cells **(B)**, B cells **(C)**, MDSCs **(D)**, and regulatory T cells (Tregs) **(E)**. Comparison of the expression of the crucial immune-checkpoint molecules between bladder cancer patients in the CLR-low group and the CLR-high group **(F–I)**. The colors represent the trend of gene expression in different samples. *p < 0.05, **p < 0.01, ***p < 0.001. The statistical differences between the two groups were compared using a Wilcox test.

TIMER (Tumor IMmune Estimation Resource, Dana Farber Cancer Institute and Harvard University, MA, USA; https://cistrome.shinyapps.io/timer/) was used to analyze associations between CLR expression and immune infiltration in the bladder microenvironment. We focused on macrophages, neutrophils, dendritic cells (DCs), CD8+ T cells, regulatory T cells, B cells, and MDSCs. A strong positive correlation was found between infiltration of CD8+ T cells, regulatory T cells, and MDSCs and expression of all four CLRs (**Figures 5B, D**, **E**). In the case of B cells, there was a medium-strength positive correlation with the expression of Dectin-1 and Dectin-2. In addition, the correlation between the expression of a range of crucial immune-checkpoint proteins and the CLRs was analyzed. The profiles of the RNA-sequencing expression and the corresponding clinical information of the 406 bladder cancer patients were downloaded from the TCGA database, and we selected sialic acid-binding Ig-like lectin 15 (Siglec15), programmed death ligand 1 (PD-L1), T-cell immunoglobulin mucin-3 (TIM-3), programmed cell death 1 ligand 2 (PD-L2), lymphocyte activation gene 3 (LAG3), T-cell immunoreceptor with Ig and ITIM domains (TIGIT), cytotoxic T-lymphocyte-associated protein 4 (CTLA-4), and programmed cell death protein 1 (PD-1) as the crucial immune-checkpoint molecules, looking at their expression values. The patients were divided into two groups based on the median transcript expression of the CLRs – the CLR-low group (*n* = 203) and the CLR-high group (*n* = 203) – and the expressions of the selected immune-checkpoint proteins were compared. We found significantly higher levels of expression of immune checkpoint proteins among patients in the CLR-high group than among patients in the CLR-low group (**Figures 5F–I**).

#### Associations between C-type lectin receptors expression and immune checkpoint blockade therapy response

**Figure 6:**
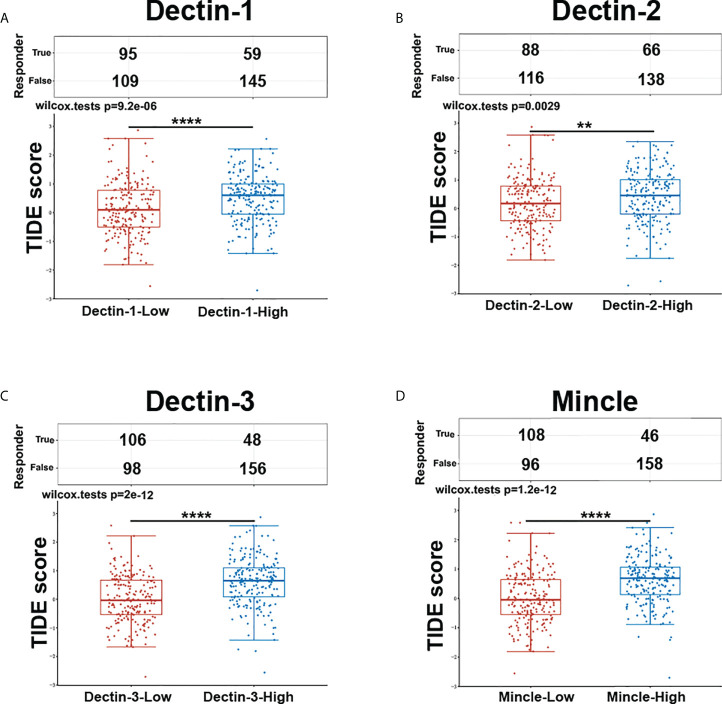
Prediction of response to ICB therapy between bladder cancer patients in the CLR-high and CLR-low groups (**A**. Dectin-1, **B**. Dectin-2, **C**. Dectin-3, **D**. Mincle). The enrolled bladder cancer patients (n = 406) were divided equally into two groups based on median transcript expression levels of CLRs. Patients in the CLR-high group had consistently higher TIDE scores than those in the CLR-low group. *p < 0.05, **p < 0.01, ***p < 0.001, ****p < 0.0001. The statistical differences between the two groups were compared using a Wilcox test.

The computational framework Tumor Immune Dysfunction and Exclusion (TIDE) (Dana Farber Cancer Institute and Harvard University, MA, USA; http://tide.dfci.harvard.edu) was developed as a tool to identify the crucial factors regulating tumor immune escape, which can be utilized as a feasible and reliable biomarker to predict ICB therapy response. The higher the TIDE score, the worse the immune evasion status, meaning that the ICB response is likely to be poorer. We utilized TIDE to evaluate the association between CLR expression and ICB response in bladder cancer. The clinical information and the RNA-sequencing data of 406 bladder cancer patients were downloaded from the TCGA database and the expression groups of the CLR genes were extracted according to expression value. Based on the median mRNA expression levels of the CLRs, the patients were further divided into two groups: the CLR-high group (*n* = 203) and the CLR-low group (*n* = 203). Both the statistical table and the scoring of immune responses are shown in **Figure 6**. Patients with high levels of expression of all CLRs displayed significantly higher TIDE scores than patients with low levels of expression, meaning that patients in the CLR-high group were at greater risk of immune dysfunction and evasion and may be insensitive to ICB therapy.

### Integration of the results of the analysis and expansion of the hypothesis

#### Potential modulatory functions on the expression of immune-checkpoint proteins by C-type lectin receptors

As shown in **Figures 5F–I**, a high level of expression of the four CLRs is significantly correlated with the upregulation of a range of immune-checkpoint ligands and proteins in the microenvironment of the bladder in bladder cancer, and they can be divided into two subgroups: those mainly expressed on tumor cells, such as PD-L1 and PD-L2, and those mainly expressed on T cells, including PD-1, CTLA-4, TIM-3, LAG3, and TIGIT. In the traditional view, immune-checkpoint expression serves as the guardian of systemic homeostasis against the overactivation of the immune system and subsequent damage to normal tissue. Nevertheless, in the context of cancer, this mechanism can be exploited by tumor cells to induce the exhaustion of T cells and inhibit their functions, eventually leading to immune escape and promoting the further deterioration of cancer. Based on the phenotypes showing a positive correlation between the expression of CLRs and a range of immune-checkpoint proteins, we discuss multiple mechanisms that CLRs may utilize to regulate the function of immune-checkpoint proteins. Moreover, we discuss in depth how this process could influence the efficacy of ICB therapy.

Multiple pathways could be utilized to control the expression of immune-checkpoint proteins, including receptors, signal molecules, transcription factors, epigenetic modulation, post-transcriptional regulation, and post-translational regulation. As with the regulation of receptor-activated signaling pathways, cytokine–receptor interaction seems to be an important mode to exert possibly crucial functions on the expression of immune-checkpoint proteins related to CLR regulation. A wide range of cytokines have been identified as key upstream factors that trigger the expression of immune-checkpoint proteins (94). For example, the γ-chain cytokines, such as IL-2, IL-7, IL-12, IL-21, and IL-15, could increase the expression of TIM-3 and PD-1 in T cells by activating the Janus kinase (JAK)-signal transducer and activator of transcription (STAT) signaling pathway following the recognition of IL-receptors (94–97). In addition, transforming growth factor beta (TGF-β), interferon alpha (IFN-α), and interferon gamma (IFN-γ) could regulate the upregulation of multiple immune-checkpoint proteins (98, 99). Notably, the anti-fungal immunity mediated by CLRs is also involved in the production of a range of functional cytokines. Signaling of Dectin-1 could induce the secretion of IL-12 following the activation of the Raf-1/NF-κB pathway, subsequently inducing the Th1 response (61). Moreover, Dectin-1 was found to be involved in the production of tumor necrosis factor α (TNF-α) in monocytes under the stimulation of fungal ligands (100). Interestingly, the positive correlation between CLR expression and the immune-checkpoint proteins is a highly coordinated phenotype, which extends across almost all the crucial immune-checkpoint proteins, as shown in **Figures 5F–I**, suggesting that there might be a pivotal upstream regulator. Chihara et al. (101) found that another immunoregulatory cytokine, IL-27, was able to drive the expression of a module of co-inhibitory immune-checkpoint proteins, including the combination of PD-1, TIM-3, LAG-3, TIGIT, and a range of atypical immune-checkpoint proteins, which is mediated by the downstream co-regulation of PR domain zinc finger protein 1 (PRDM1) and c-musculoaponeurotic fibrosarcoma (c-MAF) transcription factors. Recently, Fisher et al. (77) reported that, following infection with *O. tsutsugamushi*, the pulmonary expression of Mincle was significantly upregulated, promoting the sustained production of IL-27 and forming the pro-inflammatory immune response to infection. Furthermore, another study showed that the combined use of Dectin-1 agonist zymosan and IFN-β could enhance the expression of IL-27 in the context of multiple sclerosis (102). Although the association between CLRs and IL-27 in bladder cancer has not been fully elucidated, we can speculate that this potential pathway could be utilized by tumor cells to promote the expression of a variety of immune-checkpoint proteins and further facilitate the formation of immune resistance. We further analyzed the correlation between the expression of CLRs and the immune-checkpoint protein-regulating cytokines, specifically the correlation between the four CLRs and crucial cytokines (**Figure S1A**). The results demonstrated a significant positive correlation between the expression of these CLRs and the expression of the crucial cytokines regulating the expression of immune-checkpoint proteins. Importantly, because we paid special attention to IL-27, we also explored the relationship between the CLRs and the IL-27 downstream functional transcription factors of PRDM1 and c-MAF (**Figure S1B**). We speculated that the expression of CLRs is significantly correlated with the increase in these two transcription factors. Both the reviewed literature and our analysis suggest that we may be able to obtain more evidence that CLRs play a regulatory role in the expression of immune-checkpoint proteins, but more experiments are required to verify this hypothesis.

In addition to the immune-checkpoint receptors mainly expressed on immune cells, the immune-checkpoint ligands mainly expressed on tumor cells or macrophages are also important immunoregulators involved in carcinogenesis. Recently, Chen et al. (103) explored for the first time the relationship between the microbiota profile and PD-L1 expression in patients with non-muscle-invasive bladder cancer. There were significant differences in the composition of specific bacteria genera between the PD-L1-high group and the PD-L1-low group. This study indicated that the urogenital microbiota may be a factor affecting PD-L1 in bladder cancer patients. Regarding the mechanisms, we assumed two possible directions. One is similar to what we have discussed above: the regulation of the production of certain functional cytokines, such as IFN-γ, TGF-β, and IL-27, could increase PD-L1 mRNA expression (104), which may partly explain the mechanism of PD-L1 upregulation in the regulation of fungi–CLR interaction. The other possible direction is from the expression characteristics of CLRs, which are mainly expressed in innate immune cells, such as macrophages, dendritic cells, monocytes, and MDSCs (105, 106). It is worth noting that, in addition to tumor cells, these myeloid-derived innate immune cells can also express PD-L1 in the tumor microenvironment (107, 108). Considering the classical downstream signaling pathway following activation of CLRs, which results eventually in the activation of NF-κB, we may wonder whether this pathway or some other unknown pathways can directly promote PD-L1 mRNA transcription on CLRs activation. Surprisingly, a recent study found that administration of the agonist of another member of the CLR family – C-type lectin domain family 5 member A (CLEC5A) – resulted in increased expression by macrophages of a series of anti-inflammatory molecules, including PD-L1, which skewed the macrophages toward a pro-tumoral and anti-inflammatory phenotype (109). However, the authors did not explore the exact mechanism of this effect, and we are still uncertain whether this phenotype is regulated at the transcriptional level or by the alteration of multiple cytokines. In addition, other modulatory modes could also be utilized by CLRs to regulate immune-checkpoint proteins, such as post-transcriptional and post-translational regulation. Therefore, more experiments are required to uncover the functional mechanisms of CLRs in modulating the immune response in bladder cancer.

As shown in **Figure 6**, patients were divided into two groups according to mRNA expression of CLRs, the CLR-low and CLR-high groups, and TIDE scores were compared between the groups. TIDE scores represent the degree of immune escape by measuring the dysfunction of tumor-infiltrated cytotoxic T cells and the exclusion of tumor-infiltrated cytotoxic T cells by immunosuppressive factors. Interestingly, the TIDE scores of patients in the CLR-high group were significantly higher than those of patients in the CLR-low group, which means that high levels of expression of CLR are significantly associated with a poorer immune escape status in the tumor microenvironment in bladder cancer. In addition, high levels of expression of CLRs in bladder cancer may predict a poorer response to ICB therapy and lower survival rates after ICB therapy in bladder cancer patients. This result is consistent with the results we found in **Figure 5**, that is, high levels of expression of CLRs are positively correlated with a wide range of immune-checkpoint molecules, which indicates that the high expression state of CLRs may be an important indicator of a more immunosuppressive microenvironment. Notably, several studies have explored the relationship between intratumoral microbiota and ICB therapy efficacy. Nejman et al. (110) found significant variation in the composition of microbiota between responders and non-responders to immunotherapy from the tumor samples of melanoma patients. Moreover, a unique tumor microbiota signature was found to be closely related to better survival rates in pancreatic cancer patients (111). However, whether the alteration of the mycobiome in bladder cancer can influence ICB treatment efficacy remains unknown. The strong positivity of the expression of some immune-checkpoint molecules, such as PD-L1, PD-1, and CTLA-4, is an effective biomarker for ICB administration and can predict a positive response to ICB therapy (112–114). However, we can see from the results of the analysis that CLR expression has a tight and highly consistent positive correlation with a wide range of almost all of the crucial immune-checkpoint molecules, which suggests that high levels of expression of CLRs are linked with a highly immunosuppressive tumor microenvironment in bladder cancer. Therefore, targeting a single immune-checkpoint protein, such as PD-L1 or PD-1, may be insufficient. It may be more effective to double or even triple block the selected immune-checkpoint molecules. Moreover, as discussed above, the upstream pivotal regulator controlling the co-expression of a range of immune-checkpoint proteins may be a potential target worthy of more attention, especially IL-27. Combined inhibition of IL-27 and other immune-checkpoint molecules may be a potential choice for bladder cancer patients with high levels of expression of CLRs.

Based on the pivotal immune-modulatory functions, it has been found that the specific composition of gut microbiota has a great impact on treatment efficacy (115, 116), which has introduced a potential novel ICB synergistic therapy targeting the regulation and remodeling of the commensal microbiota, mainly through probiotic management and microbial transplantation. Recently, Gao et al. (117) found that the use of *Lactobacillus rhamnosus* probiotic could improve the gut microbiota, increasing the levels of beneficial bacteria and thus significantly improving the efficacy and responsiveness of anti-PD-1 immunotherapy in colon cancer. Moreover, transplanting the fecal microbiota from long-term responder melanoma patients to refractory metastatic melanoma patients was found to be able reverse immune resistance to anti-PD-1 immunotherapy and further optimize the efficacy of ICB therapy (41). In the fungal mycobiome, *in vivo* transplantation of specific fungi has been performed to explore its immune-regulatory roles. Wang et al. (40) performed the mono-colonization transplantation of *C. tropicalis* in germ-free mice during the inflammatory induction of colon cancer and found that this transplantation significantly accelerated tumor progression, whereas colonization with other fungi, *Saccharomycopsis fibuligera*, did not induce this phenotype. Moreover, both *in vivo* and *in vitro* experiments were performed and it was found that *C. tropicalis* can promote the polarization and activation of MDSCs. However, pathogenic or beneficial fungal species in bladder cancer have not been identified. Therefore, more research is required to determine the immune functions of the mycobiome in bladder cancer and utilize *in vivo* transplantation to identify the biological functions and the mycobiome’s impact on the efficacy of ICB treatment.

#### Possible mechanisms of regulation on the immune cells by C-type lectin receptors-mediated immunity

Within the tumor microenvironment, multiple innate and immune cells infiltrate or reside alongside the tumor cells and stromal cells. Active crosstalk between these forms a delicate, dynamic immunoregulatory network. Notably, the state of the immune microenvironment largely determines ICB therapy efficacy. According to this theory, tumor immune status can be divided into three subtypes, “immune-deserted”, “immune-excluded”, and “immune-inflamed”, depending on the degree of infiltration of cytotoxic T cells and their exhaustion state in the tumor microenvironment (118, 119). In addition to T cells, the differentiation, infiltration, and function of other immune cells in the tumor microenvironment are also key factors determining ICB therapy efficacy (120–122). Therefore, to further evaluate potential fungi- and CLR-mediated immune regulation in immune cells in bladder cancer and explore its impact on ICB therapy efficacy, we conducted a TIMER analysis to specifically explore the association between CLRs and some immune cells, as shown in **Figures 5A–E**.

Our analysis focused on three cell types, including CD8+ T cells, regulatory T cells, innate myeloid immune cells, and B cells, which are important immunomodulatory factors in the tumor microenvironment during ICB treatment. CD8+ effector T cells play a central role in triggering anti-cancer immune responses through cytotoxicity and apoptosis induction (123). Therefore, the high levels of infiltration of CD8+ T cells in the tumor microenvironment before ICB therapy are commonly viewed as a signal of improved immune activity and may predict an optimal response to ICB therapy (124). As shown in **Figure 5B**, the recruitment of CD8+ T cells has a moderate positive correlation with the expression of the four CLRs in the microenvironment of bladder cancer. On stimulation of Dectin-1 with the fungi-derived β-glucan ligands, dendritic cells would be activated and thus induce the expansion of CD8+ T cells and enhance their differentiation into cytotoxic T cells, which depends on Dectin-1/spleen tyrosine kinase (SYK) pathway activation (125). Furthermore, Hass et al. (126) confirmed that the adaptor protein CARD9 is an indispensable mediator in dendritic cells for cytotoxic T-cell induction by Dectin-1 and thereby controls tumor growth. Similar functions and mechanisms were also identified in Dectin-2 (127), Dectin-3 (128), and Mincle (129). These studies indicate that CLRs may also utilize the classical SYK/CARD9 pathway to induce the expansion of CD8+ T cells in the tumor microenvironment in bladder cancer and thus influence ICB therapy efficacy. However, CD8+ T cells are not the only determinators of anti-cancer immunity. Regulatory T cells suppress anti-tumor immune response through attenuating CD8+ T-cell functions, which are dependent on the release of multiple immunosuppressive cytokines and high levels of expression of immune-checkpoint molecules (130). Depletion of regulatory T cells by anti-PD-1 or anti-CTLA-4 agents has been found to be able to effectively strengthen the effector T-cell function and thereby synergize ICB therapy efficacy (131, 132). Interestingly, as shown in **Figure 5E**, the high levels of infiltration of regulatory T cells are strongly linked to the high levels of expression of CLRs in bladder cancer. Recently, Karnam et al. (133) utilized an *Aspergillus fumigatus* model to find that dendritic cells can promote regulatory T-cell polarization through activation of the WNT/β-catenin signaling pathway and PD-L1 induction. In addition to this non-classical pathway, Dectin-1 was also found to be able to induce Treg differentiation through SYK signaling and subsequent IL-1β secretion (134). From these studies we can conclude that CLRs are a double-edged sword in the inflammatory environment. It maintains a delicate immune equilibrium between CD8+ T cells and regulatory T cells. However, how this balance is hijacked by tumor cells in bladder cancer remains unknown. More importantly, we may need to identify which fungi drive and prime this process.

In addition to the adaptive immune cells, the innate immune cells are of great importance. Innate immunity represents the basis for all immune responses and serves as the first line of defense against invading pathogens or tumor cells. Furthermore, CLRs are mainly expressed in the innate myeloid immune cells, including dendritic cells, macrophages, and neutrophils. Therefore, we also explored the correlation between CLR expression and innate immune cells, especially myeloid cells. MDSCs are a pivotal immune cell subset with strong immunosuppressive capabilities in the tumor microenvironment. First, MDSCs can express a high load of PD-L1 in multiple cancers, including bladder cancer (135). Second, MDSCs can utilize different modulatory functions, such as reactive oxygen species (ROS) induction, arginase 1 (ARG-1) secretion, and inducible nitric oxide synthase (iNOS) production, to inhibit the immune response of natural killer (NK) cells and effectors cells (136) and promote the immunosuppressive capabilities of tumor-associated macrophages (137) and regulatory T cells (138). Consistent with these immunosuppressive capabilities, MDSCs have been found to be an effective target for ICB therapy, and the elimination or inhibition of MDSC could help optimize ICB therapy efficacy (139, 140). As we can see from **Figure 5D**, high levels of expression of the four CLRs are significantly associated with more abundant infiltration of MDSC in the bladder microenvironment in bladder cancer. Multiple studies have explored the relationship between CLRs, fungi, and MDSCs. For example, Rieber et al. (141) found that the pathogenic fungi *C. albicans* and *A. fumigatus* can induce the activation of MDSCs through the Dectin-1/SYK/CARD9 signaling pathway, which subsequently results in the attenuation of T-cell and NK-cell immune response against pathogenic infection. Notably, another study found that Dectin-1 plays an important contributing role in the tumorigenesis of aged oral squamous cell carcinoma in mice. Increased fungal burden and MDSC infiltration were observed in the tumor-loaded mice, which could be alleviated by the inhibition of Dectin-1, indicating that Dectin-1 can participate in the formation of an immunosuppressive milieu by increasing MDSC expression in oral cancer (142). Moreover, the degree of this MDSC induction effect seemed to be partly dependent on the fungal species (143), suggesting that the predominance of different fungal species may determine the degree of MDSC infiltration in bladder cancer. In addition to MDSCs, macrophages and dendritic cells are also indispensable participators in anti-cancer immunity. CLRs are mainly expressed on macrophages and dendritic cells, and we explored the relationship between the expression of CLRs and dendritic cells and macrophages. As with macrophages, we found a slightly positive correlation between the expression of CLRs and macrophages. In the context of cancer, tumor-associated macrophages played a dual role, and can be divided into two subtypes: anti-cancer M1 macrophages and pro-tumoral M2 macrophages (144). Based on the divergent immunoregulatory roles played by tumor-associated macrophages, several strategies have been developed to suppress M2 differentiation and function or promote conversion to M1 phenotype, which may help increase ICB therapy efficacy (145, 146). In pancreatic cancer, Dectin-1 was found to be able to reprogram the tumor-infiltrating macrophages into immune tolerogenic phenotypes and subsequently induce adaptive immunosuppression, which is dependent on the activation of the SYK signaling pathway (35). However, in myocardial ischemia–reperfusion (IR) injury, Dectin-1 activation on monocytes was found to promote M1 polarization, which suggests that CLRs may mediate both pro- and anti-inflammatory functions in different situations. Therefore, it is of great importance to determine how CLRs regulate the polarization of macrophages and subsequently influence adaptive immunity in bladder cancer. The immune functions of dendritic cells in the tumor microenvironment are also dependent on the varied phenotypes, which can be divided into immature dendritic cells and conventional type I dendritic cells. The immature phenotypes of dendritic cells display a disability of antigen presentation to activate the effector T cells, which further fosters an immunosuppressive milieu (147). In contrast, conventional dendritic cells can effectively activate the T-cell immune response and synergize the efficacy of ICB therapy (148). The results of our analysis show that high levels of expression of CLRs are significantly associated with higher levels of dendritic cell infiltration, which suggests that CLRs in bladder cancer may also participate in the regulation of immune response by modulating dendritic cell differentiation and function. Wang et al. (149) found that the activation of Dectin-1 on dendritic cells could induce the secretion of IL-33, which is a great anti-tumor immunity mediator. This function is dependent on the SYK/NRF-1/NF-κB signaling pathway. This study indicated that Dectin-1-mediated DC activation might be a potential target for improving ICB therapy. Similarly, another study identified that Dectin-1 activation on dendritic cells could further induce IL-9 secretion and Th9 immune response, which exerts powerful anti-cancer immunity functions (150). In addition, Dectin-2 and Dectin-3 have been found to have a similar role in fungal infection (128). However, in a multicenter study, IL-9 was found to be an immunosuppressive factor in the bladder microenvironment in muscle-invasive bladder cancer, which mainly depleted the activity of NK cells and CD8+ T cells and further induced immune evasion (151). Although it is not clear if the upregulation of IL-9 in bladder cancer is derived from CLR-mediated dendritic cell activation, we still need to acknowledge that CLR-regulated dendritic cell functions may not always be anti-tumoral, and further research is required to elucidate this issue.

In addition to the direct modulation of immune cells in the tumor microenvironment by CLRs, chemokines play an indispensable role in the regulation of immune cells in the tumor microenvironment. Immune cells function by trafficking into the tumor site and making contact with each other, a process largely dependent on a subfamily of cytokines called chemokines. Through binding to their receptors, the chemokines can be recruited to the region under the requirement of the distinct environment (152, 153). Considering the significant association that we found between CLR expression and the infiltration of various immune cells in the microenvironment, we can speculate that CLRs may also regulate the expression and function of immune cells in bladder cancer by regulating the secretion and alteration of chemokines. Surprisingly, in the analysis of differentially expressed genes (**Figures 3A–D**), we found that a range of chemokines were significantly upregulated in bladder cancer patients in the CLR-high group compared with those in the CLR-low group. Expression of the CXC subfamily chemokines (CXCL9, CXCL10, and CXCL13) and of the CC subfamily chemokine CCL18 was consistently found to be higher in bladder cancer patients in the CLR-high group than in the patients in CLR-low group. CXCL9 and CXCL10 are predominantly produced by monocytes under the stimulation of IFN-γ (154, 155) whereas CXCR3 is the main receptor bound by the ligands CXCL9 and CXCL10 (156). Notably, the expression of CXCR3 is also mainly regulated by IFN-γ (157). On recognition of CXCL9 and CXCL10, the CXCR3-induced axis will mediate the recruitment, activation, and differentiation of various immune cells and thus regulate the immune reactivity of a range of crucial immune cells, including NK cells, cytotoxic lymphocytes, and macrophages (155). Regarding immune cell differentiation, the CXCL9/10/CXCR3 axis was found to be able to regulate the Th1 response (158, 159), T-cell differentiation (158), and M2 macrophage differentiation (160). Furthermore, the inducement of Th1-cell activation and differentiation promotes anti-cancer immunity by activating NK cells, macrophages, and cytotoxic T cells (161, 162). In bladder cancer, the tumor-associated dendritic cells were found to secrete CXCL9, increasing PD-L1 expression in the tumor cells *via* the CXCR3/STAT3/AKT signaling pathway, thus resulting in the inhibition of anti-tumor adaptive immunity (163). Nevertheless, Kubon et al. (164) found that the low levels of expression of CXCL9 and PD-L1 were unfavorable prognostic factors in non-muscle-invasive bladder cancer, which suggested that the anti-tumor effect of CXCL9-mediated immune function may depend in part on the progression of tumorigenesis. In addition, CXCL10 was found to be released in large amounts in CD14+ cells after BCG induction therapy in bladder cancer patients. *In vitro* experiments verified that CXCL10 can enhance the migration and recruitment of effector NK cells and T cells following BCG-induced CXCR3 upregulation (165). Therefore, we may wonder whether CLRs can also modulate the immune response in bladder cancer through control of the CXC or CC chemokines. Guo et al. (166) demonstrated that lung-infiltrated dendritic cells and neutrophils can secrete CXCL9 and CXCL10 to recruit plasmacytoid dendritic cells to the lung in response to fungal infection of *A. fumigatus*, which is dependent on Dectin-1 activation. This study indicated that Dectin-1 could induce the production of CXC ligands to recruit immune cells, and this process may also exist in the context of cancer. However, the exact downstream signaling pathway remains unclear. As with CCL18 in bladder cancer, the biological functions seemed to be different from those of CXCL chemokines. Liu et al. (167) found that CCL18 could promote the development of bladder cancer through induction of EMT. Another study found that CCL18 could promote the invasiveness and metastasis of urothelial carcinoma through activation of the phosphatidylinositol-3-kinase (PI3K)/AKT/mammalian target of rapamycin (mTOR) signaling pathway (168). Although the relationship between CCL subfamily chemokines and CLRs is still unknown, we may suspect that the CLRs also participate in the development of bladder cancer.

To sum up, based on the results of our analysis – certain CXC and CC chemokines are significantly upregulated in bladder cancer patients with high levels of expression of CLRs and differentially expressed immune cells and immune-checkpoint molecules in the tumor microenvironment – we hypothesize that CLRs may also modulate immune functions through the regulation of chemokines in the tumor microenvironment in bladder cancer.

#### The protein–protein interactive networks centered by C-type lectin receptors and potential regulatory mechanisms

In addition to the above discussion and hypothesis, which are primarily based on the identified signaling pathway and common immune modulatory functions, we also analyzed interactions between the CLRs and other potential protein chaperones, which could be physical or functional relationships. Therefore, we utilized the STRING analysis to perform a protein–protein interaction network analysis to discover more potential functional mechanisms of CLRs and better understand the biological functions of CLRs. In **Figure 3I**, the different nodes represent different individual proteins derived from a single gene locus, and the colored lines represent protein–protein interactions with different mechanisms. First, we can see that there are multiple interactions among the four CLRs, which suggests co-expression or a synergistic effect of the four CLRs. Notably, multiple studies have illustrated a range of protein–protein interactions between CLRs. Dectin-3 and Dectin-2 can form heterodimers, which increases their capacity to recognize their ligands, α-mannans, result in stronger binding, which helps to induce greater immune responses against fungal infection (169). In addition, a fusion protein screening platform was utilized to detect a tight and direct interaction between Dectin-1 and Dectin-2, which synergically modulates the immune response during the process of allergic inflammation in the skin (170). Following these findings, more research is needed to elucidate the interactive working mechanisms of CLRs in the bladder microenvironment in bladder cancer.

Besides the internal protein–protein interactions between the CLRs, the interactions between CLRs and other proteins are also of great importance and may possess potent biological functions. Intriguingly, in the map of protein–protein interactions, we can see two toll-like receptors (TLRs). TLRs are identified as another class of pattern recognition receptors mainly expressed on the innate immune cells, such as dendritic cells and macrophages (171). By sensing the conserved ligands expressed on bacterial and fungal pathogens and the endogenous damage-associated molecules, TLRs exert crucial functions in inducing innate immunity and thereby activate the subsequent adaptive immunity in infection and cancer (172). Based on the pivotal immunoregulatory roles played by TLRs in cancer, the strategy of combining TLR agonists with immunotherapy has been identified as a promising treatment option to trigger a stronger anti-cancer immune response (173). Interestingly, we can see from the networks map that the four CLRs have tight protein–protein interactions with the TLRs TLR2 and TLR4. On stimulation of certain agonists, the activation of both TLR2 and TLR4 can trigger the myeloid differentiation primary response 88 (MyD88) signaling pathway in antigen-presenting cells, including dendritic cells and macrophages, which subsequently initiates strong inflammatory responses through production of a wide range of pro-inflammatory cytokines and expression of iNOS (174). In bladder cancer, TLR2 and TLR4 would be activated by the cell wall component of *M. tuberculosis*, further inducing the inflammatory responses and laying the theoretical foundation for the anti-cancer effect of BCG intravesical instillation therapy in bladder cancer (175). Notably, a range of elegant experiments have verified the interactions between CLRs and TLRs. Ferwerda et al. (176) found that Dectin-1 could synergize with TLR2 and TLR4, with enhanced TNF-α production in macrophages, inducing stronger innate immunity. Moreover, Yadav et al. (177) illustrated that Dectin-1 can function in cooperation with TLR2 to promote a stronger immune response from macrophages against mycobacterial infection. In addition, Dectin-1 and Dectin-2 were found to be involved in anti-cancer immune responses in bladder cancer patients treated with BCG (178). Therefore, it is of great importance to explore whether the co-regulation of TLRs and CLRs can result in a better immune response to BCG therapy and ICB therapy in bladder cancer. In addition, the upregulation of TLR4 and TLR2 was found to be positively associated with increases in PD-L1 or PD-L2 in infection and cancer (179–181). Furthermore, TLR4 has been shown to be able to drive PD-L1 overexpression in lung cancer through the ERK and PI3K signaling pathways (182, 183). Considering what we have found in our analysis on the correlation between CLRs and immune-checkpoint molecules – high levels of expression of CLRs are significantly associated with PD-L1 expression – we may speculate that the synergy effect between CLRs and TLRs may serve as an indirect pathway to induce the upregulation of PD-L1 in bladder cancer. Notably, we also found that FCER1G has a tight connection with the CLRs and their downstream factors in the network map. FCER1G represents a high-affinity immunoglobulin epsilon receptor subunit that participates in the formation of the fragment crystallizable region (FcR) of immunoglobulin (184). Through binding with the immunoglobulins, the immune cells subsequently exert crucial cellular effector functions, including phagocytosis, inflammatory responses, and immune cell activation (185). A range of studies have demonstrated the involvement of FCER1G in various cancers (184, 186, 187). Moreover, high levels of expression of FCER1G have been found to be a positive biomarker of improved response to immunotherapy in glioma (188). In the case of bladder cancer, bioinformatic analysis revealed that FCER1G is a potential key immunoregulator in the microenvironment of bladder cancer (189). Therefore, we may speculate that CLRs also modulate immune responses in cooperation with FCER1G. Liang et al. (190) found that thymic stromal lymphopoietin can induce the upregulation of FcR gamma subunit-related receptors epigenetically through demethylation of FCER1G, which subsequently induce Th2 and Th17 immune responses and further activate allergic responses. Notably, Dectin-2 was found to play a crucial role, in cooperation with FcRγ, in mediating this immune function. Thus, we may also focus on the potential interactive relationship between CLRs and FCER1G and how this interaction might influence the anti-cancer immunity in bladder cancer.

**Figure 7:**
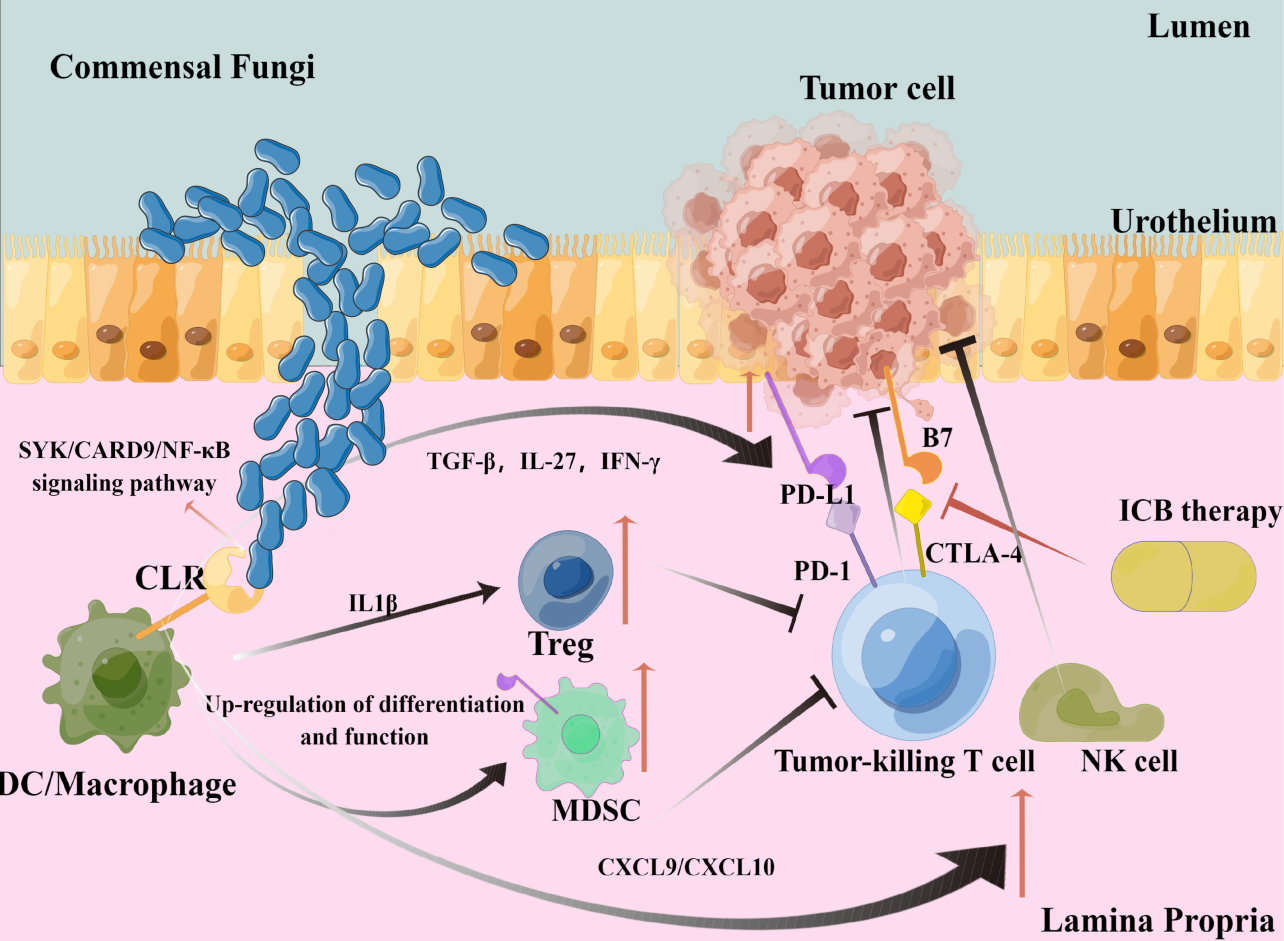
Graphical representation of our hypothesis and theory.

In conclusion, the protein–protein interaction network centered on CLRs in bladder cancer is very complex. Multiple synergistic and antagonistic interactions are interwoven with each other, making the process of CLR-mediated anti-fungal immunity more complex, and it may have further significant impacts on ICB therapy efficacy. We may need to explore more CLR-interacted targets and internal regulatory mechanisms, which may help us find more effective synergistic strategies in combination with ICB therapy in bladder cancer. Our hypothesis and theory concerning the specific cell types and pathways are summarized in **Figure 7**.

#### Non-fungal ligands recognized by C-type lectin receptors and the potential regulatory functions in bladder cancer

The common binding ligands for CLR recognition are carbohydrate ligands derived from fungi. For example, Dectin-1 mainly recognizes β-glucans, and Dectin-2, Dectin-3, and Mincle mainly recognize α-mannans (191). However, in addition to the fungal ligands, CLRs can also recognize a range of non-fungal ligands, which may also induce crucial immune responses and thus participate in the development of bladder cancer. Sin3-associated protein 130 (SAP130) is a component derived from a small nuclear ribonucleoprotein that is released from dead cells. Notably, Mincle has been found to recognize SAP130 as the damage-associated molecule pattern (DAMP) for the induction of inflammatory immune responses (192). Seifert et al. (193) demonstrated that the expression of SAP130 in pancreatic cancer can be upregulated following receptor-interacting protein (RIP)-driven cellular necroptosis, which was recognized by Mincle on the macrophages, further fostering an immunosuppressive milieu represented by the upregulation of MDSCs and M2 macrophages and a reduction in T-cell infiltration. This study indicated that non-fungal DAMP ligation with Mincle could serve as an upstream driving force to induce immune escape and oncogenesis. As for Dectin-1, one study identified vimentin as an endogenous non-fungal ligand recognized by Dectin-1 in monocytes, with activation of vimentin leading to arterial inflammation, manifested by O_2_ production, and subsequent promotion of cholesterol upregulation and lipid oxidation in atherosclerosis (194). In addition, vimentin has been identified as an oncogene in a range of cancers (195). Importantly, vimentin has been found to be an important biomarker for the process of epithelium–mesenchyme transition in bladder cancer (196, 197). Furthermore, the expression of vimentin was found to be positively correlated with indoleamine 2,3-dioxygenase 1 (IDO-1), which exerts crucial regulatory functions on T-cell functions in bladder cancer (198). Therefore, Dectin-1 may also modulate T-cell function through vimentin activation in bladder cancer, but further experiments are required to verify this hypothesis. Moreover, the non-fungal ligands recognized by CLRs also include viral ligands. Recently, Dectin-2 expressed on dendritic cells was found to be a key sensor for influenza hemagglutinin, which facilitates the induction of the immune response during influenza infection (199). Moreover, other kinds of viruses have been found to be recognized by CLRs and thus initiate inflammatory responses (200–202). Because the application of oncolytic virus therapy in cancer has been another major milestone, following the success of ICB therapy, and has achieved surprising efficacy in multiple cancers (203, 204), we may focus on CLRs as novel potential targets in oncolytic virus therapy in bladder cancer. Furthermore, it has also been found that Dectin-1 can recognize the N-glycan structure derived from tumor cells, that is, tumor-associated molecule patterns (TAMPs), thus triggering anti-tumor immune responses (205). A wide range of TAMPs have been identified in bladder cancer (206), which suggests that TAMPs may be another possible non-fungal ligand for CLR activation. Overall, in addition to the classical fungal ligands, non-fungal ligands deserve our attention for their great capabilities in inducing immune responses.

#### The site of mycobiome-immune interactions in the bladder

To more precisely illustrate the process of anti-fungal immunity mediated by CLRs in bladder cancer, we need to further discuss the immune interactions among the mycobiome, CLRs, and immune cells from an anatomical perspective. The urothelium, commonly referred to as the mucosal layer of the bladder, presents the greatest degree of impenetrability (207). This layer forms the first line of defense against any potentially threatening pathogens and toxins. Abundant immune cells, including dendritic cells, macrophages, monocytes, T cells, and NK cells, reside in the submucosa and lamina propria (208–210). Therefore, when the urothelium is intact, there are few opportunities for the fungi colonizing the urothelium to come into contact with the resident immune cells, which maintains homeostasis and the immune equilibrium of the bladder. However, in bladder cancer, this immune-balanced state can be disrupted and subsequent mycobiome immune interactions can be activated. Although this process has not been clearly modeled before, we can utilize similar models to model this process. Based on the growth of the primary tumor, we speculate that the division between the Ta or Tis stage and the ≥ T1 stages is largely determined by the site of the immune interaction. A tumor stage of ≥ T1 means that the tumor mass has spread to the lamina propria or further, which can provide more direct anatomical channels for the contact between the mycobiome and bladder-residing immune cells, especially CLR-expressing immune cells. Previous studies have shown that, in infection of uropathogenic *Escherichia coli* in the bladder, the superficial urothelial cells fall off, thus depleting the infected cells, along with the intracellular bacteria. However, this process also allows the bacteria to invade deeper under the urothelium, where they can interact with the resident immune cells and induce subsequent immune responses (211). Moreover, Wang et al. (33) found that the decrease in Dectin-3 expression can impair the phagocytic capabilities of the gut-residing macrophages and significantly upregulate the fungal burden of *C. tropicalis*, which further aggravates the severity of dextran sulfate sodium (DSS)-induced colitis. It should be noted that this process is dependent on damage to the normal intestinal mucosa and impairment of the tissue repair system. The authors further demonstrated that a defect in CARD9 expression can impair the ability of dendritic cells and macrophages to kill invading commensal fungi, which can lead to the accumulation of MDSC and promote the development of colon cancer (40). Combining these findings, we can conclude that the impairment of the intact mucosal barrier is indispensable to mycobiome immune interactions, which mainly occur in the submucosa and lamina propria. We hypothesize that this process may be aggravated in T1–4 bladder cancer, when the urothelium is badly damaged by the invasive tumor mass. Nevertheless, for tumors at the Ta or Tis stage, pre-existing bladder inflammation or mucosal injury due to bladder catheterization may also provide opportunities for mycobiome immune interactions.

Recently, Fu et al. (212) discovered a novel pro-tumoral mechanism that depends on intratumoral bacteria. The tumor-resident intracellular bacteria can enhance the metastatic capabilities of cancer cells through the reorganization of the actin cytoskeleton and the promotion of mechanical stress resistance. This study suggests that the low biomass of the intratumoral microbiota could exert crucial biological functions in the process of carcinogenesis. Despite the lack of information on the existence and composition of the intratumoral microbiota, especially fungal species, we hypothesize that, if intratumoral fungi exist, then the tumor-infiltrated immune cells would also interact actively with the intracellular mycobiome and their expressed ligands.

Notably, the discussion above focuses on the fungi with a direct attachment to epithelial cells, whereas the anatomical and functional characteristics of the bladder determine that the urine in the bladder can be another source of urinary fungal microbiota. First, the effectiveness of bladder instillation of BCG in bladder cancer shows that the bacterial components or other immunogenic substances in the free-flowing liquid in the bladder possess sufficient tissue permeability to infiltrate the tumor or urothelium tissue and induce the inflammatory responses that can kill the tumor cells (213). As discussed, emerging evidence challenges the traditional idea that urine is sterile, and the bacterial burden was found to be significantly upregulated in the urine samples of bladder cancer patients compared with non-neoplastic patients. More importantly, increased bacterial richness is positively associated with the risk of progression and recurrence (46). Notably, in addition to microbes, microbial fragments and DNA can also be detected in the urine and may serve as the interactive ligands for CLR recognition. To conclude, the site of interaction between the mycobiome and immune cells is typically the submucosa or intratumoral region, but more experiments are required to model this process more precisely.

#### The potential association between C-type lectin receptors expression and fungal mycobiome distribution in bladder cancer

Based on our hypothesis, the interaction between CLRs and the fungal mycobiome is the dominant component of the antifungal immune response in bladder cancer and the subsequent anti-tumor immune response and determines the efficacy of ICB therapy. Therefore, it is of great importance to elucidate the association between CLR expression and the mycobiome. Consider, for example, Dectin-1, which is a primary sensor of commensal or pathogenic fungi in humans (78). A decrease in or complete absence of the Dectin-1 signaling pathway has been reported to lead to increased susceptibility to fungal infection and related diseases (214). Moreover, polymorphism or deficiency of Dectin-1 has been found to induce abnormal activation of immune responses and to alter the fungal mycobiome (215, 216). Notably, the deficiency of Dectin-3 or the CARD9/SYK axis in dendritic cells and macrophages resulted in a fungal dysbiosis represented by an increase of *C. tropicalis*, which subsequently led to an accumulation of MDSC, thus promoting tumor progression (33, 40). In summary, adequate innate immune cell infiltration with sufficient CLRs expression is important in the homeostasis of mucosal fungi, and absent or low levels of expression of specific CLRs can result in the abnormal expression of certain commensal fungi and subsequently exert pro-tumoral immune functions.

Interestingly, a significant increase in Dectin-1 was found in bladder cancer tumor tissues compared with normal tissues at the transcriptional level (**Figure 2A**). This phenomenon raises a new question: is the increase in Dectin-1 expression due to the infiltration of immune cells in turn due to the fungal mycobiome or is it due to the endogenous expression of Dectin-1 in cancer cells? Liu et al. (217), in mice models, investigated the alteration of the expression of Dectin-1 and its downstream adaptor proteins during the host response to pulmonary infection with *A. fumigatus* and found that stimulation of fungal conidia could significantly increase the expression of Dectin-1 in myeloid cells. Moreover, another study has shown that, during the process of fungus-induced inflammation, taurine chloramine, which is an inflammatory product derived from activated neutrophils, can upregulate the expression of Dectin-1 on the macrophages, thereby increasing their phagocytic capacity (218). Collectively, the expression of Dectin-1 can be upregulated directly by modulation of the fungal mycobiome or indirectly by fungus-induced inflammation-derived products. Given the possibility of the existence of a fungal mycobiome in the bladder, the significantly upregulated expression of Dectin-1 may be due to the enhancement of the function and infiltration of immune cells under the modulation of the mycobiome.

Chiba et al. (205) found that Dectin-1 expressed on immune cells can also sense the N-glycan structure of tumor cells and thus activate anti-cancer immune responses, which indicates that the fungal ligand may not be the only stimulator for CLR activation and that TAMPs may also be ligands at CLRs and regulate their expression or activation. Notably, a range of tumor-derived glycans have been found in bladder cancer, including the glycans Lewis X (LeX), Sialyl Lewis X (SLeX), Sialyl Tn (STn), and N-glycolyl GM3 (NGcGM3) (206). Therefore, the upregulation of Dectin-1 in bladder cancer may also be under the regulation of these TAMPs without the participation of the fungal mycobiome, but the interactions between CLRs and TAMPs in bladder cancer must be researched further.

Notably, in another study, the authors explored the tumoral expression of Dectin-1 and found that Dectin-1 has a predominant expression in the tumor cells of clear cell renal cell carcinoma (ccRCC) and that high levels of expression of Dectin-1 are associated with a worse prognosis (219). This suggests that CLRs, in addition to exerting their immune functions as a result of their expression on immune cells, may also mediate the regulation of tumorigenesis through non-classical approaches. Therefore, another possibility arises: significantly increased expression of Dectin-1 in tumor tissue in bladder cancer may result in the endogenous overexpression of Dectin-1 on the tumor cells. However, despite the verification of the tumoral expression of Dectin-1, the concrete biological functions and signaling mechanisms are still unknown. Therefore, further research is required to elucidate the modulatory functions exerted by the tumor cell expressed CLRs.

## Discussion and future directions

From the limited available sequencing data on the urinary mycobiome, we can see that the commensal fungi residing in the bladder are highly diverse and heterogeneous, providing a complex crosstalk network interacting with the host immune system. Although we can draw no definitive conclusion about the relationship between the urinary mycobiome and urological diseases, research on similar organs and immune status may be relevant. The organ whose commensal microbiota has been most widely studied is the gastrointestinal tract, especially the gut. Wang et al. (40) found that in CARD9 deficiency the commensal fungus *C. tropicalis* can pass through the mucosal barrier and induce MDSC infiltration, which ultimately facilitates colon tumorigenesis. CARD9 is the most commonly required adaptor for the activation of innate immune responses on recognition of fungal ligands from CLRs (220, 221). Our results show that the commensal fungi and CLR-mediated immune surveillance jointly maintain a delicate mucosal immune balance in the gut. This relationship resembles a scale, with both sides in balance, and strengthening or weakening of either side can cause the collapse of the whole system. Similarly, on the basis of the existence of the commensal fungi in the bladder, we found that members of the CLR family are actively expressed in the bladder at both mRNA and protein levels. Among all of the CLRs, Dectin-1 displayed the greatest role in enrichment, and transcriptional expression of Dectin-1 and Dectin-2 was at significantly higher levels in tumor tissue than in normal tissue (**Figure 2**). IHC showed that the level of expression of both Mincle and Dectin-3 varies greatly among individuals. However, it is apparent that in normal urothelial tissue CLRs are mostly expressed on the mucosa, which may serve as an immune barrier and protect against potential fungal invasion. Further studies are required to determine the dominant fungi during the carcinogenesis of bladder cancer. In addition, dynamic alterations in spatial distribution and internal components should be closely monitored to further understand the process of CLR–fungi crosstalk.

Anti-pathogen immune responses can be considered biphasic, comprising primary innate immunity and secondary specific adaptive immunity (222). On recognition of fungal ligands or carbohydrate antigens, the CLRs, which are mainly expressed on the cell surface of myeloid cells, trigger the downstream signaling pathways and activate a range of transcription factors, including NF-κB. The subsequent production of various pro-inflammatory factors can induce the recruitment of the leukocytes and activate innate immunity, including phagocytosis (191, 223). In addition, with the activation of innate immunity, phagocytic cells can engulf fungal pathogens or infected cells and prime them into fungi-derived antigen protein to present to T cells, which subsequently induce Th1, Th17, and T follicular helper (TFH) immune responses (30, 224). Despite the indirect antigen presentation activation of adaptive immunity, CLRs can also be directly expressed on lymphocytes, such as B cells, NK cells, and γδ T cells, to induce pro-inflammatory responses (225, 226). Although we cannot conclude from this that the CLR-related biological functions are caused by the mycobiome in the bladder, which participate in the formation of urological malignancy, we may at least suspect that, during the tumorigenesis of bladder cancer, the CLRs exerted some immune-regulatory functions, which may be a contributory factor for anti-cancer immunity in bladder cancer and should not to be ignored. After performing the gene-related pathway analysis, we explored the association between CLR expression and crucial immune cell infiltration. For innate immunity, we focused on myeloid phagocytic cells. We found that the expression of all CLRs had a median positive correlation with dendritic cell infiltration and a weak positive correlation with macrophage infiltration.

Future studies should address three areas. The first is the specific fungal community and compositional alterations in different stages of bladder cancer, which may help us to find the most crucial functional fungi in immune regulatory networks in bladder cancer. The second is interactions between the commensal fungi and CLRs in bladder cancer and the exact mechanisms. *In vitro* and *in vivo* experiments may help us to further understand this process, leading to the development of more effective targeting agents. The third is the pathological process that occurs in bladder cancer patients in the real world. This can be achieved by using advanced gene-sequencing techniques and by conducting a range of clinical trials.

## Methods

### Analysis of C-type lectin receptor mRNA and protein expression levels

To elucidate the expression of CLRs in normal and malignant bladder tissue, we utilized UALCAN (University of Alabama at Birmingham Cancer data analysis; University of Alabama at Birmingham, Birmingham, AL, USA; http://ualcan.path.uab.edu/) to compare the transcriptional levels in normal tissue and primary tumor tissue. The data were derived from TCGA database (normal tissue, *n* = 19; primary tumor tissue, *n* =  8). Clinical information of the enrolled bladder cancer patients could be downloaded from UCSC Xena (University of California, Santa Cruz, Santa Cruz, CA, USA; https://tcga.xenahubs.net). To observe and analyze the expression levels and location of CLR family receptors in normal bladder tissue and bladder cancer tumor tissue, we used the HPA to acquire IHC images of CLR (Dectin-3 and Mincle) staining in both types of tissues.

### Differentially expressed gene acquisition and pathway enrichment prediction

Data relating to a total of 408 bladder cancer patients were extracted from the TCGA database. To screen DEGs based on the differentiated expression of CLRs, patients were divided into two subgroups: a CLR-low group (G1) and a CLR-high group (G2), based on the median expression of CLR. We performed a hierarchical clustering analysis of mRNAs, and constructed volcano plots of significant DEGs based on the fold-change values and adjusted *p*-values. Red dots represent overexpressed mRNAs and blue points down-expressed mRNAs with statistical significance. To predict potential downstream pathways and possible biological functions, we performed GO analysis of significantly upregulated mRNAs. The biological process (BP), cellular component (CC), and molecular function (MF) of potential targets were clustered based on ClusterProfiler package in R version 3.18.0 (The R Foundation for Statistical Computing, Vienna, Austria).

### Immune infiltration analysis

To further determine the correlations between CLRs and immune cell infiltration in the bladder cancer tumor microenvironment, we utilized TIMER, analyzing the correlations between the transcriptional expression of CLRs and macrophages, dendritic cells, and neutrophils. In addition, we used specific markers of other immune cells, such as CD8+ T cells, MDSCs, and B cells, for comparison with the expression of CLRs to comprehensively explore the correlations between CLRs and immune infiltration. CD8+ T cells were represented by the expression of CD8A and CD8B. B cells were represented by the expression of CD19, CD79A, and CD209. MDSCs were represented by the expression of ITGAM and CD33.

### Immune-checkpoint blockade therapy response prediction analysis

To evaluate the relationship between CLR expression and immunotherapy efficacy in bladder cancer patients, we downloaded data from the TCGA database. To divide the enrolled patients into two groups, the CLR-low group and the CLR-high group, data on the expression of CLR genes were extracted. The TIDE algorithm was utilized to predict the potential response to immune checkpoint blockade therapy (227).

### Statistical analysis

All statistical comparisons between the two groups were performed with the Student’s *t*-test. Spearman’s correlation was utilized to determine the correlation between quantitative variables without a normal distribution. A *p*-value of < 0.05 was considered statistically significant.

## Data availability statement

The original contributions presented in the study are included in the article/**Supplementary Material**. Further inquiries can be directed to the corresponding authors.

## Author contributions

RY and HG conceived and developed the research idea. THL extended and systemically analyzed the feasibility of the scientific hypothesis and wrote the paper. TYL, YP, and ZZ participated in the discussion and provided crucial components of the hypothesis. XX and YZ participated in the discussion and collected data. SZhan, SZhou, and WZ polished the language and revised the manuscript. All authors read and approved the final manuscript.

## Funding

This work was supported by the National Natural Science Foundation of China (81772727, 82172691, and 81772710) and Nanjing Science and Technology Development Key Project (YKK19011).

## Acknowledgments

The authors express sincere appreciation to all members of the Immunology and Reproductive Biology Laboratory, Medical School, Nanjing University, and the M3 laboratory of the School of Life Sciences, Nanjing University, for their generous help and inspiring discussions. In addition, we express great thanks to Professor Tingting Wang and Professor Yayi Hou, et al., from Nanjing University Medical School, and Junliang Zhang from the Department of Philosophy, Nanjing University, for their generous knowledge sharing and inspiration. The figure of the graphical representation of our hypothesis and theory was drawn and generated by Figdraw (www.figdraw.com).

## Conflict of interest

The authors declare that the research was conducted in the absence of any commercial or financial relationships that could be construed as a potential conflict of interest.

## Publisher’s note

All claims expressed in this article are solely those of the authors and do not necessarily represent those of their affiliated organizations, or those of the publisher, the editors and the reviewers. Any product that may be evaluated in this article, or claim that may be made by its manufacturer, is not guaranteed or endorsed by the publisher.

## Supplementary material

Supplementary Figure 1 Heatmap of the correlation between CLRs and multiple cytokines **(A)**. The abscissa and ordinate represent the genes, respectively. Colors represent different correlation coefficients (blue represents positive correlation; red represents negative correlation); the darker the color, the stronger the relation. **p* < 0.05, ***p* < 0.01. The correlation between the expression of the CLRs and the two key downstream transcription factors: PRDM1 and c-MAF **(B)**.
