# Supplementary figures and images for: Antifungal immunity mediated by C-type lectin receptors may be a novel target in immunotherapy for urothelial bladder cancer
Source: Front Immunol. 2022 Sep 5;13:911325. doi: 10.3389/fimmu.2022.911325 (PMC9483128; doi:10.3389/fimmu.2022.911325)

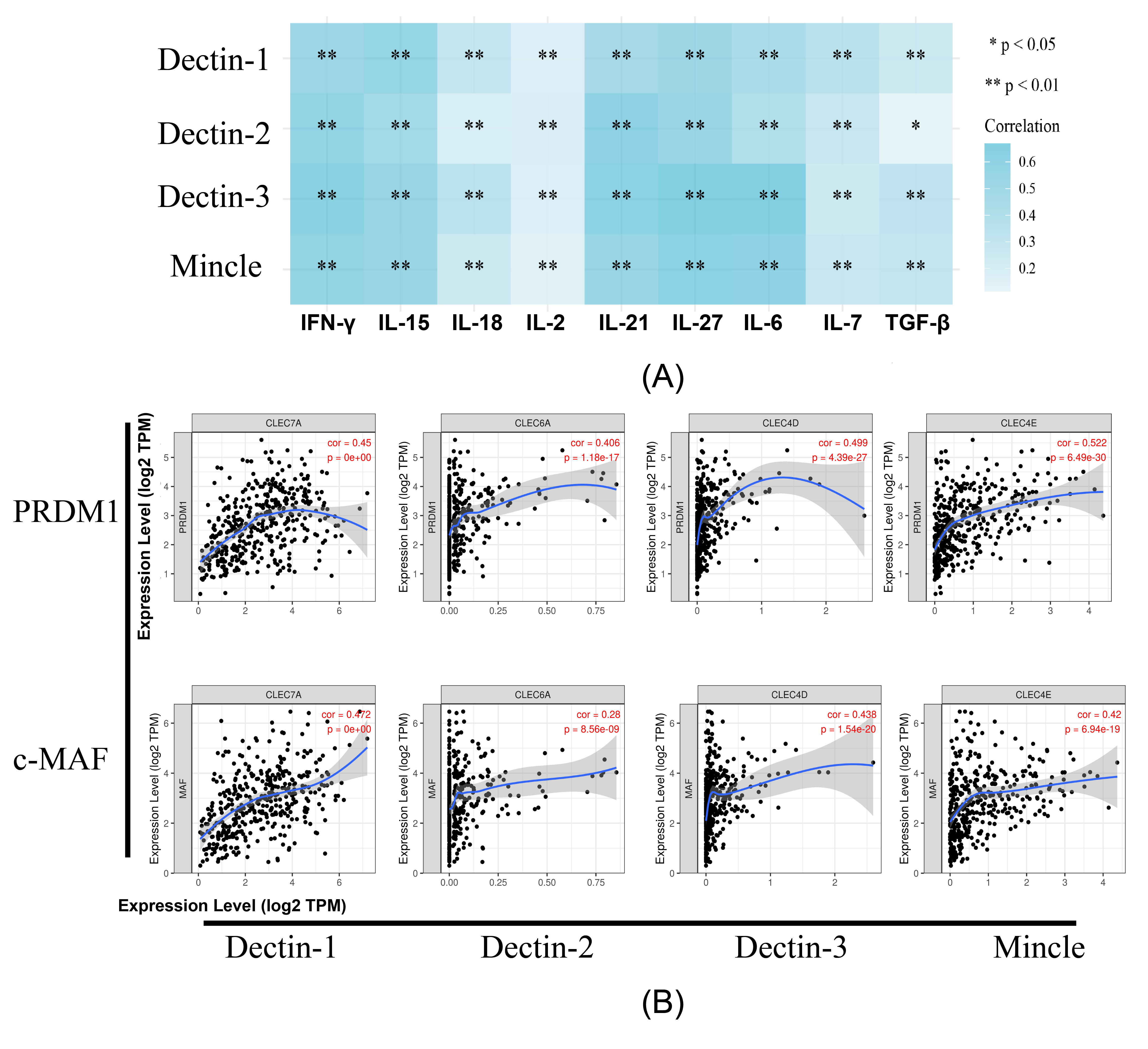

Supplement: Supplementary Figure 1 — Heatmap of the correlation between CLRs and multiple cytokines (A). The abscissa and ordinate represent the genes, respectively. Colors represent different correlation coefficients (blue represents positive correlation; red represents negative correlation); the darker the color, the stronger the relation. *p < 0.05, **p < 0.01. The correlation between the expression of the CLRs and the two key downstream transcription factors: PRDM1 and c-MAF (B). [file Image_1.tif]
